# The analgesic effect of total intravenous anaesthesia with propofol versus inhalational anaesthesia for acute postoperative pain after hepatectomy: a randomized controlled trial

**DOI:** 10.1186/s12871-023-02063-7

**Published:** 2023-04-03

**Authors:** Stanley S. C. Wong, Fengfeng Wang, Timmy C. W. Chan, C. W. Cheung

**Affiliations:** 1 Department of Anaesthesiology, School of Clinical Medicine, Li Ka Shing Faculty of Medicine, The University of Hong Kong, Hong Kong, China; 2 Department of Anaesthesiology, Queen Mary Hospital, Hong Kong, China

**Keywords:** Propofol TIVA, Pain scores, Morphine consumption, Adverse effects, Hepatectomy

## Abstract

**Background:**

Postoperative pain control can be challenging in patients undergoing hepatectomy. A previous retrospective study on hepatobiliary/ pancreatic surgeries showed better postoperative pain control in patients who received propofol TIVA. The aim of this study was to determine the analgesic effect of propofol TIVA for hepatectomy. This clinical study has been registered at ClinicalTrials.gov (NCT03597997).

**Methods:**

A prospective randomized controlled trial was performed to compare the analgesic effect of propofol TIVA versus inhalational anaesthesia. Patients aged between 18 and 80 years old with an American Society of Anesthesiologist (ASA) physical status of I-III scheduled for elective hepatectomy were recruited. Ninety patients were randomly allocated to receive either propofol TIVA (TIVA group) or inhalational anaesthesia with sevoflurane (SEVO group). Perioperative anaesthetic/analgesic management was the same for both groups. Numerical rating scale (NRS) pain scores, postoperative morphine consumption, quality of recovery, patient satisfaction and adverse effects were evaluated during the acute postoperative period and at 3 and 6 months after surgery.

**Results:**

No significant differences were found for acute postoperative pain scores (both at rest and during coughing) and postoperative morphine consumption between TIVA and SEVO groups. Patients given TIVA had lower pain scores with coughing at 3 months after surgery (*p* = 0.014, and FDR < 0.1). TIVA group was associated with better quality of recovery on postoperative day (POD) 3 (*p* = 0.038, and FDR < 0.1), less nausea (*p* = 0.011, and FDR < 0.1 on POD 2; *p* = 0.013, and FDR < 0.1 on POD 3) and constipation (*p* = 0.013, and FDR < 0.1 on POD 3).

**Conclusion:**

Propofol TIVA did not improve acute postoperative pain control compared to inhalational anaesthesia in patients who underwent hepatectomy. Our results do not support the use of propofol TIVA for reducing acute postoperative pain after hepatectomy.

## Background

Propofol is a commonly used intravenous anaesthetic drug. It can be used in total intravenous anaesthesia (TIVA) to maintain general anaesthesia throughout surgery without using inhalational anaesthetics [1]. Some of the advantages for propofol-based TIVA include less post-operative nausea and vomiting, better postoperative psychomotor function, and faster recovery [2]. Propofol has analgesic and anti-hyperalgesic effects [3]. Moreover, propofol inhibits the production of pain-mediated pro-inflammatory cytokines IL-1β, IL-6, and TNF-α [4]. It also inhibits the NMDA receptor, which plays an important role in the pain transmission process [5, 6]. Clinical studies that have compared the postoperative analgesic effect of propofol TIVA versus inhalational anaesthesia do not show consistent results. While a number of clinical studies have found better pain control with propofol TIVA, others have reported no significant difference [7–10]. One meta-analysis showed that propofol TIVA was associated with a statistically significant, but small reduction in pain scores and opioid consumption at 24 h after surgery [11]. On the other hand, another meta-analysis found no clinically significant benefit with propofol TIVA when a conservative *p*-value was used to account for heterogeneity [12].

The postoperative analgesic effect of propofol TIVA is perhaps influenced by the types of surgery and accompanying analgesic regimes [13]. Liver resection is a major surgery associated with moderate to severe pain. Postoperative pain control after liver resection can be challenging, owing to concerns with epidural analgesia in patients with potential coagulopathy/thrombocytopaenia and limitation of systematic analgesic options in patients with liver dysfunction. Good postoperative pain control is important to minimize suffering, improve patient satisfaction, reduce morbidity, enhance recovery and decrease the risk of chronic postoperative pain [14]. Previous results from a retrospective study demonstrated that propofol TIVA was associated with reduced pain scores with coughing and reduced postoperative opioid consumption when compared to inhalational sevoflurane after hepatobiliary and pancreatic surgeries [15]. In the retrospective study, a heterogenous variety of surgeries were included: hepatectomy, radiofrequency ablation of the liver and pancreatic resection. Propofol TIVA could potentially contribute to reducing acute postoperative pain after hepatectomy, but this has not been studied in a prospective randomized controlled trial.

A prospective randomized controlled trial is the reasonable next step to determine propofol TIVA’s analgesic effect for hepatectomy. The main objective of this study is to investigate the effect of propofol TIVA on acute pain intensity and opioid consumption after hepatectomy when compared to inhalational anaesthesia. We hypothesize that propofol TIVA would reduce postoperative pain scores and opioid consumption.

## Methods

This study was conducted at Queen Mary Hospital in Hong Kong, China. The study was approved by the Institutional Review Board of The University of Hong Kong/Hospital Authority Hong Kong West Cluster (UW 18–176), and registered at clinicaltrial.gov prior to patient recruitment on 24/07/2018 (NCT03597997). All methods were carried out in accordance with relevant guidelines and regulations. Written informed consent was obtained from all patients participating in the trial.

This study was prospective, double-blinded randomized controlled, conducted in accordance to the CONSORT guideline. All methods were carried out in accordance with Declaration of Helsinki. Patients aged between 18 and 80 years old with an American Society of Anesthesiologist (ASA) physical status of I-III scheduled for elective hepatectomy (left or right hepatectomy, segmentectomy, or wedge resection) were eligible. Exclusion criteria included the following: 1) known drug allergy to propofol, opioids, and non-steroidal anti-inflammatory drugs (NSAIDs) or COX-2 inhibitors, paracetamol, ketamine; 2) alcohol or drug abuse; 3) impaired renal function, defined as preoperative serum creatinine level over 120 µmol/L; 4) impaired or retarded mental state; 5) body mass index (BMI) > 35 kg/m^2^; 6) history of chronic pain; 7) pregnancy; 8) local infection; 9) history of psychosis; 10) delirium; 11) chronic opioid user; or 12) patient refusal [16].

Eligible patients were approached in the general ward before the operation for recruitment. The anaesthetic options were explained, and the patient was recruited into the study if s/he agreed. The patients were then randomized into one of two groups: i) SEVO group, anaesthetized by inhalational anaesthesia using sevoflurane; or ii) TIVA group, anaesthetized using propofol total intravenous anaesthesia. Patients recruited for hepatectomy were stratified in randomization. A computer-generated random sequence was used to select the allocation order, which was concealed in opaque envelopes, and opened at the time of intervention administration. Patients were not aware of the types of anaesthesia they received. A separate blinded investigator assessed the patients after the operation. The anaesthetist providing general anaesthesia was aware of the allocation, but s/he was not involved in data collection. Assessment was done at the general ward. Fasting for patients started at midnight before operation [16]. Premedication was not prescribed.

On arrival to the operation theatre, a 20- or 22-gauge intravenous cannula was inserted. Standard monitoring with pulse oximeter, non-invasive blood pressure, and three lead electrocardiograms was applied prior to induction. For patients in the SEVO group, general anaesthesia was induced with propofol 1.5-3 mg/kg, remifentanil 0.5-1mcg/kg, and rocuronium 0.6-1 mg/kg or atracurium 0.5 mg/kg given intravenously (IV). Sevoflurane, air and oxygen was used for maintenance of general anaesthesia. FiO_2_ was be kept between 35–50%. Bispectral Index (BIS) monitoring was applied and the level of anaesthesia was titrated to maintain a BIS value of between 40–60. Intravenous remifentanil infusion between 0.1–0.2 mcg/kg/min was given during surgery and titrated to provide optimal haemodynamic parameters. Ondansetron 4 mg IV was given 30 min before the end of surgery. Sevoflurane and remifentanil infusion were switched off at the end of the procedure. Reversal of muscle relaxation was obtained with neostigmine 50mcg/kg IV and atropine 20mcg/kg IV after the operation. Patients were transferred to the post-anaesthetic care unit (PACU) for monitoring for at least 30 min. The anaesthetic and analgesic protocol for patients in the TIVA group was the same as the SEVO group. The only difference was that the induction and maintenance of general anaesthesia was performed using propofol total intravenous anaesthesia. Sevoflurane was not used, and oxygen and air were given. Target controlled infusion (TCI) with modified Marsh effect site model (Fresenius Kabi) was used for induction and maintenance of general anaesthesia. Level of anaesthesia was titrated to produce a BIS value of between 40–60. During induction of general anaesthesia, remifentanil 0.5-1mcg/kg, and rocuronium 0.6-1 mg/kg or atracurium 0.5 mg/kg were given IV. Remifentanil infusion was given as per SEVO group [16].

For both groups of patients, morphine sulphate at a bolus dose of 0.1 mg/kg IV was given before skin incision. Additional 0.1 mg/kg of morphine sulphate IV could be given in divided doses when the surgery continued for more than 2 h at the discretion of the attending anaesthetist. Ketamine 0.5-1 mg/kg IV was given before skin incision. Patients received local wound infiltration with up to 2 mg/kg of levobupivacaine during wound closure [16]. This was administered by the surgeon under direct visualization of the tissue layers using a 22G needle attached to a 10 ml syringe containing 0.5% levobupivacaine. The needle was directly inserted into the subdermal and musculofascial layers throughout the wound length. Local anaesthetic was injected after negative aspiration at each location along the wound.

Resting pain scores were checked every 5 min in the PACU. Morphine sulphate at a dose of 2 mg IV was given every 5 min until the numerical rating scale (NRS) pain score was less than 4/10. Respiratory rate, oxygen saturation, Ramsay sedation scores, blood pressure and heart rate were monitored every 5 min in the PACU. Patient controlled analgesia (PCA) morphine was connected to the patient once the NRS pain score was less than 4/10. The following parameters were set for the PCA morphine machine: bolus 1 mg of intravenous morphine sulphate with each patient demand, lockout duration of 5 min, and maximum dose limit of 0.1 mg/kg of morphine sulphate per hour. When the patient resumed fluid diet in the ward on POD 0, regular oral dihydrocodeine was prescribed at a dose of 30 mg three times a day for two days. Afterwards, dihydrocodeine was given as needed. Breakthrough pain was treated by intramuscular/subcutaneous morphine 0.1 mg/kg every 4 h as needed starting from POD 0 [16]. Pain related parameters such as NRS pain scores at rest and during coughing, cumulative PCA morphine doses and side effects were recorded every 4 h.

PCA morphine was given for at least 2 days. It was stopped on POD 2 if NRS pain scores during coughing were less than 4/10 and morphine consumption was low. PCA morphine was continued if NRS pain scores during coughing were equal or greater than 4. After discontinuation of PCA morphine, NRS pain scores (at rest and during coughing) and the dose and frequency of rescue analgesia used were recorded daily until patient discharge. Patient satisfaction with analgesia, where 0 corresponded to the least satisfaction, and 10 corresponded to the most satisfaction, was assessed on POD 1 [16]. Quality of recovery 9 (QoR-9) was evaluated on POD 1 and POD 3, which contains 9 questions to assess postoperative changes in emotion, well-being, social function and physical disability [17].

Various outcomes were also assessed at 3 and 6 months after surgery. This included the presence or absence of pain, and NRS pain scores at rest and with coughing [16]. Patient satisfaction with analgesia, health related quality of life (assessed using the 12-Item Short-Form (SF-12) Health Survey [18]) and psychological status (assessed using the Hospital Anxiety and Depression Scale (HADS) questionnaire) were also evaluated.

### Statistical analysis

The primary outcome was the NRS pain score during coughing on POD 1. Our previous retrospective study on hepatobiliary and pancreatic surgery had shown that the mean (SD) of NRS pain score during coughing on POD 1 for TIVA group was 4.30 (1.99) [19]. To detect a difference in NRS pain score of 1.3 out of 10 with a significance level of 0.05 at power of 80%, 37 patients per group were needed. An NRS pain score difference of 1.3 was chosen because this has been regarded as the value corresponding to a minimally clinically significant change in acute pain intensity [20]. The sample size was estimated using the methods described for clinical trials [21]. To consider for possible dropouts, 45 patients were recruited into each group, giving a total of 90 patients.

For patient demographics and intraoperative analgesic consumption, continuous data were analysed using independent-samples t-test or Mann–Whitney U test, and categorical data were analysed using Pearson Chi-square test or Fisher’s Exact test. HRQOL domains for SF-12, satisfaction with analgesia and psychological condition were compared with Mann–Whitney U test. The standardized pain scores both at rest and during coughing were expressed as area under curve (AUC) weighted by the corresponding time interval. The weighted AUC was equivalent to a time weighted average of the pain scores for the specified time interval, and was of the same scale as the NRS (0–10). The difference in the standardized pain scores between the TIVA and SEVO groups were compared using the Mann–Whitney U test, and expressed as median (interquartile range). The cumulative PCA morphine consumption at 24, 48 and 72 h after the surgery between the two groups was also compared using the Mann–Whitney U test. Incidence of adverse effects was compared using the Chi-square test or Fisher’s exact test as appropriate. IBM SPSS Statistics Version 27.0 (IBM Corp. USA) was used to analyse the above data. False discovery rate (FDR) is the expected proportion of the rejected null hypotheses that are actually true, and it is a recommended alternative in health-related studies [22, 23]. Benjamini–Hochberg false discovery rates (FDRs) were applied to correct for multiple-hypothesis, and a *p*-value < 0.05 with an FDR value < 0.1 was regarded as statistically significant [24].

## Results

**Fig. 1:**
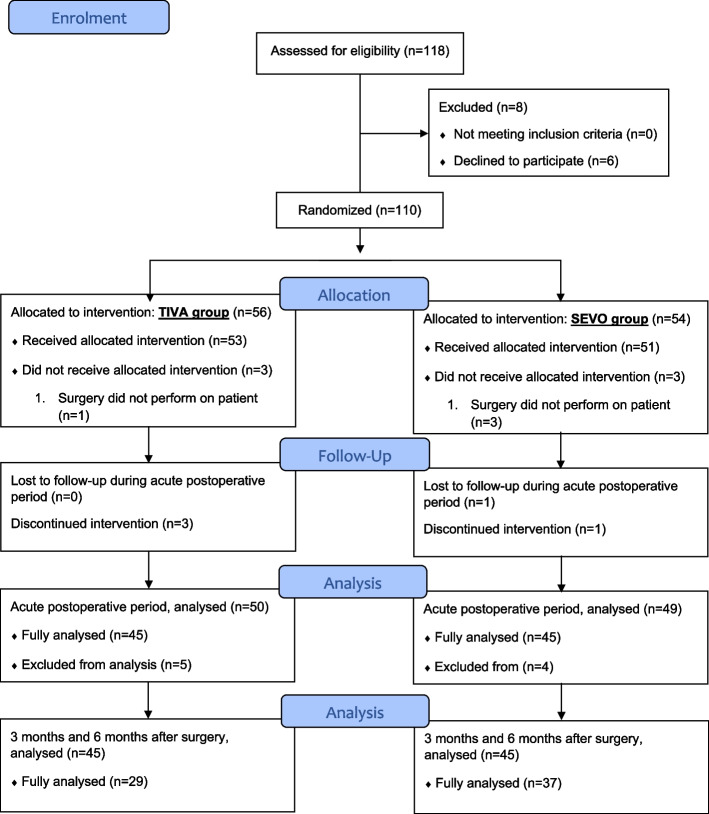
Flow diagram of patients involved in this study

Ninety patients in total (45 in each group) completed the study, which was conducted from August 2018 to July 2021. One hundred and eighteen patients were assessed for eligibility, and eight were excluded. Six patients declined to participate and two patients did not join due to medical decisions. A total of 110 patients were randomly assigned to undergo anaesthesia, either with propofol TIVA (*n* = 56) or inhalational sevoflurane (*n* = 54). Six patients in the TIVA group and five patients in the SEVO group were excluded. A total of 99 recruited patients completed the study for primary outcome measure. Five patients in the TIVA group and four patients in the SEVO group were excluded due to insufficient data, and the results of the remaining 90 patients were used for analysis. At 3 and 6 months after surgery, 16 patients in the TIVA group and 8 patients in the SEVO group were lost to follow up, leaving 29 patients in the TIVA group ad 37 patients in the SEVO group for analysis (Fig. 1: Flow diagram of patients involved in this study).

There were no significant differences between TIVA and SEVO groups in patient characteristics and intraoperative parameters, including age, body weight, gender, ASA status, duration of surgery, duration of anaesthesia, intraoperative morphine consumption, intraoperative ketamine consumption, intraoperative remifentanil consumption and blood loss after FDR correction (Table 1).

**Table 1:** Patient characteristics and intraoperative parameters

	TIVA (*n* = 45)	SEVO (*n* = 45)	*P*-value (FDR)
Age (year)	55.98 ± 13.59	60.18 ± 13.23	0.141
Body weight (kg)	67.5 (59.35–75.05)	63 (56.6–72.5)	0.154
Gender			0.612
Female	11 (24.4%)	9 (20%)	
Male	34 (75.6%)	36 (80%)	
ASA			0.186
1	5 (11.1%)	1 (2.2%)	
2	23 (51.1%)	29 (64.4%)	
3	17 (37.8%)	15 (33.3%)	
Duration of surgery (min)	273 (189.5–372.5)	308 (165.5–387)	0.738
Duration of anaesthesia (min)	334 (234.5–450)	376 (251–446)	0.678
Intraoperative morphine used per body weight (mg.kg-1)	0.105 (0.091–0.150)	0.119 (0.095–0.161)	0.36
Intraoperative remifentanil (bolus) used per body weight (mg.kg-1)	0.001 (0–0.002)	0 (0–0.001)	0.04 (> 0.1)
Intraoperative remifentanil-IV (infusion) used per body weight (µg.kg min-1)	0.10 (0.10–0.15)	0.10 (0.08–0.15)	0.893
Intraoperative Ketamine used per body weight (mg.kg-1)	0.489 (0.439–0.511)	0.423 (0.299–0.500)	0.068
Intraoperative morphine (mg)	8 (6-9)	8 (6-10)	0.845
Intraoperative remifentanil (bolus) (mg)	0.07 (0–0.1)	0 (0–0.068)	0.037 (> 0.1)
Intraoperative Ketamine (mg)	30 (25-35)	25 (20-30)	0.027 (> 0.1)
Levobupivacaine (Bolus) (mg)	50 [0–100]	100 [15–100]	0.409
Levobupivacaine per body weight (Bolus) (mg.kg-1)	1.29 [0–1.67]	1.39 [0.76–1.64]	0.371
Blood loss (ml)	387.5 (162.5–962.75)	500 (75–870)	0.99
Types of liver surgery			0.012 (> 0.1)
Hepatectomy (Right or left)	17 (37.8%)	27 (60%)	
Segmentectomy	3 (6.7%)	8 (17.8%)	
Wedge resection	20 (44.4%)	9 (20%)	
Others	5 (11.1%)	1 (2.2%)	

*p*-value (FDR) refers to *p*-value (false discovery rate), and a significant difference was identified if *p* < 0.05 and FDR < 0.1; continuous data were analyzed by Independent-samples t test or Mann–Whitney U test; categorical data were analyzed by Pearson Chi-square test or Fisher’s Exact test; values in median [Interquartile range], mean ± standard deviation, or N (%)

**Fig. 2:**
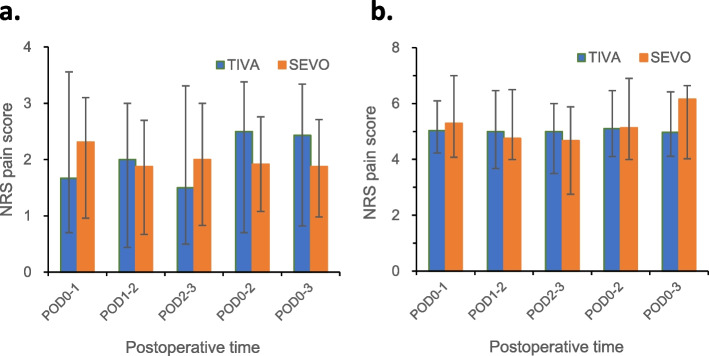
The weighted AUC for postoperative NRS pain scores between patients anaesthetized by TIVA and SEVO. **a** Pain scores at rest. **b** Pain scores during coughing. Data were analyzed by Mann–Whitney U test. Values expressed in median [Interquartile range]. AUC: area under the curve; NRS: numerical resting scale

No significant differences were identified in AUC NRS pain scores between patients in the TIVA and SEVO groups, both at rest (at POD 0–1, POD 1–2, POD 2–3, POD 0–2 and POD 0–3) and during coughing (at POD0-1, POD 1–2, POD 2–3, POD 0–2 and POD 0–3) (Table 2 and Fig. 2: The weighted AUC for postoperative NRS pain scores between patients anaesthetized by TIVA and SEVO). Moreover, there were no significant differences in postoperative PCA morphine consumption at 24, 48 and 72 h after the surgery between the two groups (Table 3). We also stratified the patients into laparoscopic and open surgery for analysis for postoperative NRS pain scores and morphine consumption. No significant differences in pain scores (both at rest and with coughing) and postoperative opioid consumption were found between TIVA and SEVO groups when laparoscopic and open surgeries were analyzed separately. The QoR score in the TIVA group was significantly higher than the SEVO group on POD 3 (*p* = 0.038, and FDR < 0.1) (Table 4). There were no differences in the incidence of adverse effects on POD 1. However, patients in SEVO group were more likely to experience nausea on POD 2 (*p* = 0.011, and FDR < 0.1) and POD 3 (*p* = 0.013, and FDR < 0.1), and constipation (*p* = 0.013, and FDR < 0.1) on POD 2. There was a statistically significant increase in overall adverse effects on POD 2 in the SEVO group compared to the TIVA group (*p* = 0.007, and FDR < 0.1) (Table 5). No significant difference was identified for patient satisfaction on POD 1.

**Table 2:** The weighted AUC values for the standardized pain scores between patients anaesthetized by TIVA and SEVO at rest and during coughing

	TIVA (*n* = 45)	SEVO (*n* = 45)	*P*-value
***Pain scores at rest***			
POD0-1	1.67 [0.70–3.56]	2.31 [0.96–3.10]	0.652
POD1-2	2.00 [0.44–3.00]	1.88 [0.67–2.70]	0.866
POD2-3	1.50 [0.50–3.31]	2.00 [0.83–3.00]	0.880
POD0-2	2.50 [0.70–3.38]	1.92 [1.08–2.76]	0.723
POD0-3	2.43 [0.82–3.34]	1.88 [0.98–2.71]	0.554
***Pain scores during coughing***			
POD0-1	5.03 [4.23–6.10]	5.29 [4.08–7.00]	0.519
POD1-2	5.00 [3.67–6.46]	4.75 [4.00–6.50]	0.951
POD2-3	5.00 [3.50–6.00]	4.67 [2.75–5.88]	0.478
POD0-2	5.10 [4.10–6.46]	5.13 [4.00–6.90]	0.859
POD0-3	4.97 [4.11–6.42]	5.16 [4.02–6.64]	0.982

Weighted AUC indicates weighted area under curve; values in median [Interquartile range]; data were analyzed by Mann–Whitney U test

**Table 3:** Postoperative analgesic morphine consumption

	TIVA (*n* = 45)	SEVO (*n* = 45)	*P*-value
Morphine (cumulative-24 h)	15.00 [5.50–23.25]	12.00 [6.00–19.50]	0.630
Morphine (cumulative-48 h)	25.00 [11.25–39.00]	22.00 [12.00–31.00]	0.531
Morphine (cumulative-72 h)	26.25 [11.75–39.75]	22.00 [12.00–29.00]	0.556

Values in median [Interquartile range]; data were analyzed by Mann–Whitney U test

**Table 4:** Quality of Recovery (QoR) score

	TIVA (*n* = 45)	SEVO (*n* = 45)	*P*-value (FDR)
QoR score (POD 1)	13.00 [12.00–14.00]	13.00 [11.00–15.00]	0.679
QoR score (POD 3)	16.00 [14.00–17.00]	15.00 [13.00–16.00]	*0.038 (0.076)

^*^p < 0.05 and FDR < 0.1, indicating the significant difference; values in median [Interquartile range]; data were analyzed by Mann–Whitney U test

**Table 5:** Postoperative adverse effects

	TIVA (*n* = 45)	SEVO (*n* = 45)	*P*-value (FDR)
***Postoperative Day 1***			
Nausea	10 (22.2%)	15 (33.3%)	0.239
Vomiting	1 (2.2%)	1 (2.2%)	1
Dizziness	5 (11.1%)	4 (8.9%)	1
Pruritus / Itchiness	6 (13.3%)	4 (8.9%)	0.502
Wound infection	2 (4.4%)	0 (0%)	0.494
Urinary retention	7 (15.6%)	9 (20.5%)	0.547
Confusion	0 (0%)	0 (0%)	–-
Constipation	22 (48.9%)	25 (56.8%)	0.454
Presence of adverse effects (Postoperative Day 1)	32 (71.1%)	36 (81.8%)	0.234
***Postoperative Day 2***			
Nausea	3 (6.7%)	12 (26.7%)	*0.011 (0.044)
Vomiting	0 (0%)	2 (4.4%)	0.494
Dizziness	3 (6.7%)	1 (2.2%)	0.616
Pruritus / Itchiness	6 (13.3%)	5 (11.1%)	0.748
Wound infection	1 (2.4%)	0 (0%)	1
Urinary retention	2 (4.8%)	4 (10.5%)	0.416
Confusion	0 (0%)	0 (0%)	–-
Constipation	16 (38.1%)	25 (65.8%)	*0.013 (0.035)
Presence of adverse effects (Postoperative Day 2)	23 (54.8%)	33 (82.5%)	*0.007 (0.056)
***Postoperative Day 3***			
Nausea	3 (7%)	11 (27.5%)	*0.013 (0.091)
Vomiting	0 (0%)	2 (5%)	0.229
Dizziness	3 (7%)	0 (0%)	0.242
Pruritus / Itchiness	5 (11.6%)	3 (7.5%)	0.714
Wound infection	0 (0%)	0 (0%)	–-
Urinary retention	1 (2.4%)	5 (12.8%)	0.101
Confusion	0 (0%)	0 (0%)	–-
Constipation	12 (28.6%)	19 (48.7%)	0.062
Presence of adverse effects (Postoperative Day 3)	18 (42.9%)	26 (66.7%)	0.032 (> 0.1)

^*^p < 0.05 and FDR < 0.1, indicating the significant difference; data were analyzed by Chi-square test or Fisher’s exact test; values in N (%)

At 3 months after surgery, TIVA group was associated with lower pain scores compared to SEVO group during coughing (*p* = 0.014, and FDR < 0.1), but there was no difference at rest (Table 6). There were no differences in pain scores at rest and during coughing at 6 months after surgery (Table 6). There were no differences in SF-12 scores, HADS scores and patient satisfaction at 3 and 6 months after surgery (Tables 7 and 8).

**Table 6:** Postoperative pain scores between TIVA and SEVO groups at rest and during coughing for 3 and 6 months

	TIVA (*n* = 29)	SEVO (*n* = 37)	*P*-value (FDR)
***Postoperative (3 months)***			
Pain score at rest	0.00 [0.00–2.50]	1.00 [0.00–2.00]	0.574
Pain score during coughing	0.00 [0.00–1.00]	1.00 [0.00–3.00]	*0.014 (0.057)
***Postoperative (6 months)***			
Pain score at rest	0.00 [0.00–1.00]	0.00 [0.00–2.25]	0.306
Pain score during coughing	0.00 [0.00–0.00]	0.00 [0.00–2.00]	0.237

^*^p < 0.05 and FDR < 0.1, indicating the significant difference; Values in median [Interquartile range]; data were analyzed by Mann–Whitney U test

**Table 7:** Post-operation (Quality of life-SF-12 v2)

	TIVA (*n* = 29)	SEVO (*n* = 37)	*P*-value
***Postoperative (3 months)***			
Physical Functioning	57.06 [49.19–57.06]	57.06 [41.32–57.06]	0.462
Role Physical	57.46 [49.00–57.46]	57.46 [49.00–57.46]	0.892
Bodily Pain	57.73 [48.71–57.73]	57.73 [48.71–57.73]	0.991
General Health perceptions	57.69 [47.75–57.69]	57.69 [47.75–57.69]	0.538
Vitality	58.90 [49.07–68.74]	49.07 [49.07–58.90]	0.087
Social Functioning	48.01 [39.11–56.90]	56.90 [39.11–56.90]	0.419
Role Emotional	56.28 [45.89–56.28]	56.28 [45.89–56.28]	0.693
Mental Health	64.21 [52.74–64.21]	64.21 [41.26–64.21]	0.283
Physical Composite Score (PCS)	55.57 [44.55–57.62]	52.99 [46.77–56.06]	0.649
Mental Composite Score (MCS)	55.54 [51.29–62.38]	54.51 [47.09–61.40]	0.449
***Postoperative (6 months)***			
Physical Functioning	57.06 [41.32–57.06]	57.06 [49.19–57.06]	0.899
Role Physical	57.46 [46.89–57.46]	53.23 [49.00–57.46]	0.333
Bodily Pain	57.73 [53.22–57.73]	57.73 [48.71–57.73]	0.746
General Health perceptions	57.69 [52.72–57.69]	57.69 [47.75–57.69]	0.618
Vitality	58.90 [49.07–68.74]	58.90 [49.07–68.74]	0.629
Social Functioning	56.90 [39.11–56.90]	48.01 [39.11–56.90]	0.420
Role Emotional	56.28 [51.09–56.28]	56.28 [45.89–56.28]	0.857
Mental Health	64.21 [49.87–64.21]	64.21 [52.74–64.21]	0.410
Physical Composite Score (PCS)	55.57 [44.16–56.52]	53.09 [46.20–56.13]	0.494
Mental Composite Score (MCS)	58.00 [49.72–62.23]	59.70 [50.08–62.38]	0.732

Values in median [Interquartile range]; data were analyzed by Mann–Whitney U test

**Table 8:** Post-operation (Satisfaction with analgesia, and psychological condition with the hospital anxiety and depression)

	TIVA (*n* = 29)	SEVO (*n* = 37)	*P*-value
***Postoperative (3 months)***			
Satisfaction	10.00 [8.00–10.00]	9.00 [8.25–10.00]	0.408
Anxiety score	0.00 [0.00–2.50]	1.00 [0.00–4.00]	0.173
Depression	0.00 [0.00–3.00]	0.00 [0.00–2.00]	0.645
***Postoperative (6 months)***			
Satisfaction	10.00 [9.00–10.00]	10.00 [8.00–10.00]	0.109
Anxiety score	0.00 [0.00–3.00]	2.00 [0.00–4.00]	0.113
Depression	0.00 [0.00–3.00]	0.00 [0.00–3.00]	0.878

Values in median [Interquartile range]; data were analyzed by Mann–Whitney U test

## Discussion

There were no differences in overall acute postoperative analgesia after hepatectomy in terms of NRS pain scores and postoperative PCA morphine consumption between patients who received propofol TIVA and inhalational sevoflurane. Patients in the TIVA group had lower pain scores with coughing at 3 months after surgery. Propofol TIVA was associated with reduced nausea and constipation, as well as better quality of recovery (QoR) on POD 3. The analgesic effect of propofol TIVA for acute postoperative pain control is not certain. A previous meta-analysis showed a small reduction in postoperative pain intensity and opioid consumption with propofol TIVA [25]. Another meta-analysis found no difference in analgesia when a more conservative *p*-value was applied to account for heterogeneity [12]. The analgesic benefit of propofol TIVA is likely to be influenced by the types of surgical procedure and accompanying analgesic regimes.

Postoperative pain control after hepatic surgery can be challenging to manage. Hepatic surgery is associated with upper abdominal wounds, which results in more severe postoperative pain when compared to lower abdominal wounds, especially with coughing [26]. Patients with large upper abdominal wounds usually require more analgesics including opioids. Patients with liver cirrhosis may also have problems with drug metabolism and coagulopathy/thrombocytopaenia, which would limit the use of epidural analgesics and other systematic analgesic drugs (e.g. opioids and non-steroidal anti-inflammatory drugs). Dose of opioids may have to be adjusted after hepatic surgery due to impaired metabolism after liver resection, particularly for those with pre-existing liver dysfunction [27]. Therefore, it is important to evaluate whether the use of propofol TIVA for general anaesthesia would reduce pain after hepatic surgery, because it is a straightforward anaesthetic technique that can be used in patients with liver diseases. In this study, we found that propofol TIVA did not improve acute postoperative analgesia. Therefore, the choice of propofol TIVA for hepatic surgery should be based on its effect on other factors and outcomes. Patients in the TIVA group had lower pain scores with coughing at 3 months after surgery, suggesting that it may reduce chronic pain after hepatectomy. Chronic post-surgical pain is a significant clinical problem that can result in prolonged patient suffering, reduced quality of life and impaired functioning [28]. Propofol TIVA has previously been shown to reduce post-thoracotomy pain at 3 and 6 months after surgery, despite having no effect on acute postoperative pain scores [29]. Propofol inhibits NMDA receptor activity [5, 30], which may lead to reduced chronic pain. However, a larger sample size is needed to validate the current findings.

Patients in the TIVA group had reduced nausea and constipation, and better quality of recovery on POD 3. The quality of recovery (QoR) score is a measure of the health-related quality of life after surgery and anaesthesia [17]. A good QoR was important in producing a positive patient feeling during the recovery process [31], and worse QoR scores have been associated with longer duration of hospital stay [32]. Our results showed better QoR scores in the TIVA group on POD 3. Postoperative pain intensity is one of the factors that influence QoR, but other factors also contribute. Propofol TIVA has been associated with better QoR in a meta-analysis of randomized controlled trials, and the main improvement was in “physical comfort”, “emotional status”, “psychological support” and “physical independence” [33]. This suggests that propofol TIVA can improve QoR in patients undergoing hepatectomy irrespective of postoperative acute pain control. Propofol TIVA was associated with significantly less nausea and constipation compared to inhalational anaesthesia. Propofol TIVA has been known to reduce nausea and vomiting compared to inhalational anaesthesia [11, 34]. Our results showed reduced nausea but no differences in vomiting. In this study protocol, nitrous oxide was not used and all patients received prophylactic anti-emetics. As a result, the incidence of vomiting in both groups of patients was low. Since propofol TIVA was not associated with significant opioid sparing effects in this study, the difference in nausea and constipation was unlikely related its analgesic effects.

In a previous retrospective case–control study, propofol TIVA was associated with lower acute postoperative pain scores and postoperative opioid consumption after hepatobiliary and pancreatic surgeries [19]. However, our current results showed no differences for hepatectomy. This was the case for both open and laparoscopic surgery. There are various possible explanations for this. The types of surgeries studied in the two clinical studies were not identical. The previous retrospective study also included other surgeries such as pancreatic resection and radiofrequency ablation of the liver, in addition to hepatectomy. Another reason for the lack of analgesic benefit may be attributed to the use of ketamine for all patients undergoing hepatectomy in the current study. Ketamine produces analgesia by inhibiting the NMDA receptors, which is also one of the key analgesic mechanisms of propofol. Therefore, the effect of propofol could perhaps have been masked by the effect of ketamine. In addition, retrospective studies are more prone to bias, and positive results in retrospective studies may not be replicated in more robust prospective randomized controlled trials.

There were some limitations in this randomized controlled trial. The first limitation was a relatively small sample size. The sample size was calculated based on our previous retrospective study on hepatobiliary and pancreatic surgery, which was not only restricted to hepatectomy [19]. A larger sample size may have been required to detect a statistically significant difference in analgesic outcomes. However, any statistically significant difference detected may have been small and lack clinical significance. The sample size was even smaller at 3 and 6 months due to loss of follow up, and therefore may be insufficiently powered to detect significant differences for longer-term secondary outcomes. Nevertheless, the primary aim of this study was to investigate the effect of propofol TIVA for acute postoperative analgesia. The second limitation was the analgesic regime. The application of thoracic epidural analgesia has been advocated for hepatectomy [35]. However, it is not routinely used due to concerns with potential bleeding and coagulopathy/thrombocytopaenia, and it may not provide superior analgesia compared to PCA morphine [36]. Therefore, PCA morphine rather than epidural analgesia was used in our study. Nevertheless, our findings may not be applicable to patients who receive epidural analgesia for hepatectomy.

In conclusion, propofol TIVA did not improve postoperative pain scores or reduce opioid consumption after hepatectomy when compared to inhalational anaesthesia. Propofol TIVA was associated with better QoR on POD 3 and lower pain scores with coughing at 3 months after surgery. It was also associated with a lower incidence of nausea and constipation, which is not likely related to propofol’s analgesic effect. Our results do not support the use of propofol TIVA for the purpose of reducing acute postoperative pain after hepatectomy when used together with ketamine. However, other outcomes such as effects on adverse events and quality of recovery should be considered when choosing between general anaesthetic techniques.

## Data Availability

The datasets used and/or analysed during the current study are available from the corresponding author on reasonable request.

## Abbreviations

ASA: American Society of Anesthesiologist
AUC: Area under curve
BIS: Bispectral Index
BMI: Body mass index
COX-2: Cyclooxygenase-2
FDRs: False discovery rates
FiO2: Fraction of inspired oxygen
HADS: Hospital Anxiety and Depression Scale
HRQOL: Health-related quality of life
IV: Intravenously
NMDA: N-methyl-D-aspartate
NRS: Numerical rating scale
NSAIDS: Non-steroidal anti-inflammatory drugs
PACU: Post-anesthetic care unit
PCA: Patient controlled analgesia
POD: Postoperative day
QoR-9: Quality of recovery 9
SD: Standard deviation
SEVO: Sevoflurane
SF-12: 12-Item Short-Form
TCI: Target controlled infusion
TIVA: Total intravenous anesthesia
